# Study on the Influence of Friction and Wear Properties of High-Speed Rail Brake Materials under Humidity Environment and Temperature Conditions

**DOI:** 10.3390/ma16041610

**Published:** 2023-02-15

**Authors:** Siyuan Ding, Meixian Zhang, Yiding Ou, Lei Ma

**Affiliations:** Key Laboratory of Fluid and Power, Machinery of Education, School of Mechanical Engineering, Xihua University, Chengdu 610039, China

**Keywords:** high-speed rail brake material, humidity, high temperature, surface damage, pin disc test

## Abstract

A multi-functional friction and wear testing machine was used to test the pin disk wear of high-speed railway brake friction material under different disk temperatures (20 °C, 100 °C, and 200 °C) and different ambient humidities (55%, 95%). The test results show that the change in the disk temperature and different ambient humidities have significant effects on the frictional wear performance of the high-speed railway brake material. Under the conditions of 20 °C, 100 °C and 200 °C, the instantaneous friction coefficient and wear rate of the brake material decreased as the ambient humidity increased. The different ambient humidity caused severe surface damage to the brake materials, but the damage mechanisms were dramatically different. At constant temperature, the higher the ambient humidity, the lower the maximum equilibrium temperature of the disc.

## 1. Introduction

Transportation infrastructure plays an irreplaceable basic role in the development of the national economy. Each change and upgrade is an important feature of rapid economic change [1]. In 2021, the national railway operating mileage reached 151,000 km, an increase of 53,000 km compared to the end of 2012. In the process of transportation, a high-speed railway maintains a series of outstanding advantages such as low energy consumption, low pollution, high efficiency, high safety and high comfort, which provides a strong guarantee of the development of the national economy and people’s lives [2,3,4]. The construction of high-speed railways is one of the important strategic infrastructure projects in China and the most important component of China’s modern transportation system [5,6]. With the development of China’s high-speed railway and the improvement of railway transportation capacity, the abnormal wear of brake discs under extreme environmental conditions [7,8,9], such as: high temperature, low temperature, high humidity, high cold, and especially high humidity has been widely examined by scholars at home and abroad. The brake disc is one of the core components of braking in high-speed trains [10]. The level of braking technology is one of the key factors that determine the safety, comfort and stability of high-speed trains, so the study of brake discs is particularly important [11,12]. As the speed of high-speed trains continues to increase, the standard of braking performance is gradually improved, and braking technology is constantly updated in the development of high-speed trains [13]. As a major high-speed rail development country, it is necessary for China to master core braking technology.

With the rapid development of high-speed trains, they have now spread to all parts of the world, and while high-speed trains enter different regions, the different environmental problems faced by different regions have also become a pertinent research topic. There is no doubt that external factors such as the environment have a huge impact on the braking performance of the brake disc, and this influence directly determines the service life of the brake disc [14,15]. Foreign scholars, Eriksson [16] and others, studied the ambient air humidity as a variable through tribological tests, and the results showed that air humidity had a significant impact on the friction coefficient and wear of friction materials. Their influence was extremely critical. When the humidity in the air is 20~80%, the influence of humidity on the friction coefficient has a certain limit, but when the humidity is greater than 80%, the friction coefficient will increase with the increase in humidity. The research results show that in the process of brake friction, the medium water will form a fine “water film” on the friction surface, and this water film will greatly reduce the friction coefficient of the brake disc in the process of braking. The size of the water flow speed can also directly affect the braking performance, and in the braking process of the water condition, water lubrication and dry friction can be clearly distinguished. Hongyu Sun [17] et al. studied the friction and wear behavior of high-speed train braking materials at high temperature (25 °C, 200 °C, 400 °C, 600 °C), and the results showed that they had high friction coefficients at 25 °C and 400 °C, but the two temperature wear types were different. Kang-jie Rong [18] et al. studied the wear of wheel–rail brake materials in high temperature and humidity environment. The results show that humidity has a significant impact on the wear behavior of brake materials. In high-temperature and low-humidity environments, fatigue wear is the main wear mode. With the increase in humidity, abrasive wear becomes more and more serious, and gradually becomes the main form of wear. Peng Zhang [19] et al. also studied the tribological properties of high-speed railway brake materials in the background of high temperature. The results show that with the increase in temperature, the friction film will change from a single structure of mixed layer to a double-layer structure, and the wear mechanism will also change from abrasive wear to adhesion wear.

In short, researchers only study high-speed rail brake materials from the perspective of a single variable (humidity or temperature), and they rarely combine the effects of the two factors to study them. Therefore, it is necessary to study the tribological properties of brake disc brake pads of high-speed trains in a high-temperature and humidity environment.

In this study, the self-developed multi-functional friction and wear testing machine will be used to combine the influencing factors of temperature and humidity to perform the pin disc friction and wear test. The following four aspects of the damage mechanism and friction and wear properties of the material were studied: friction coefficient, wear rate, surface plane morphology and disk surface temperature increase. Through systematic and comprehensive study of the friction and wear mechanism of high-speed train materials in high temperature and humidity environment, this research provides important theoretical support and technical guidance which will ensure the safety, stability and reliability of high-speed trains traveling at high temperature and high humidity in China.

## 2. Test Details

### 2.1. Brake Discs and Brake Pads

**Figure 1:**
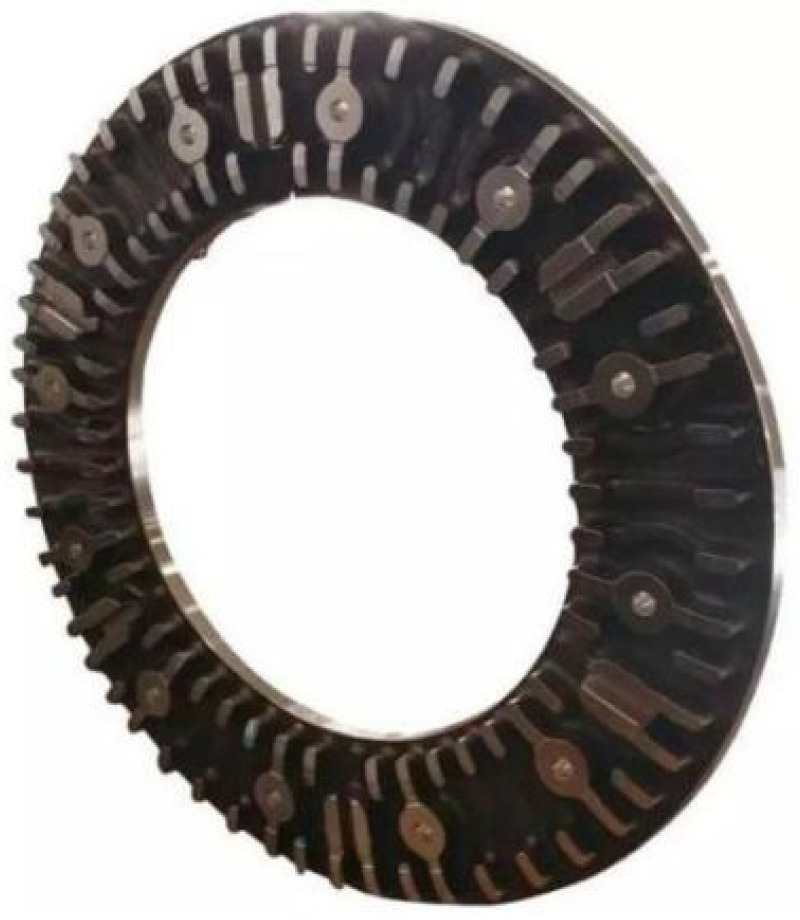
Brake disc material [4].

**Figure 2:**
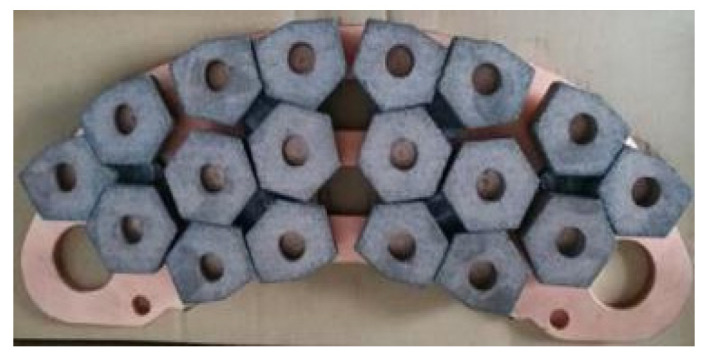
Brake pad material [4].

The brake disc and brake pad test materials used in this study are taken from the high-speed rail EMU site (CRH380) of China EMU, and the physical drawings of the brake disc and brake pad are shown in Figure 1 and Figure 2, respectively. After consulting the relevant information, it can be seen that the main component of the brake disc material is special forged steel material, and the main structure is slat-like martensite, because slat-shaped martensite has high strength, high hardness and good toughness [20,21]. The brake pad material is a copper-based powder metallurgy material, which has the advantages of high strength, good rigidity, good plasticity, oxidation resistance and corrosion resistance. In order to accurately explore the structural composition of the brake disc/brake pad material used in this study, the composition of chemical elements of forged steel brake disc material and copper-based powder metallurgy material was detected and analyzed using an EDS spectrometer and copper-based powder metallurgy material, and the test results are shown in Table 1.

### 2.2. Sample Preparation

**Figure 3:**
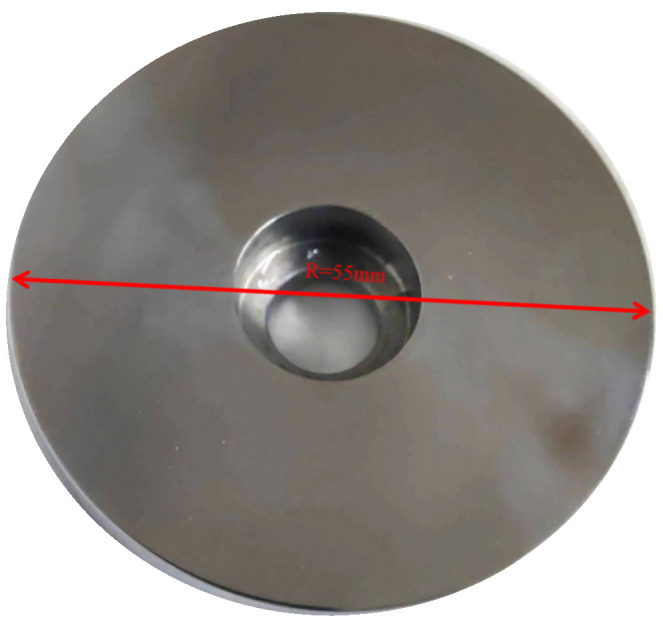
Physical diagram of the structure of the disc specimen.

**Figure 4:**
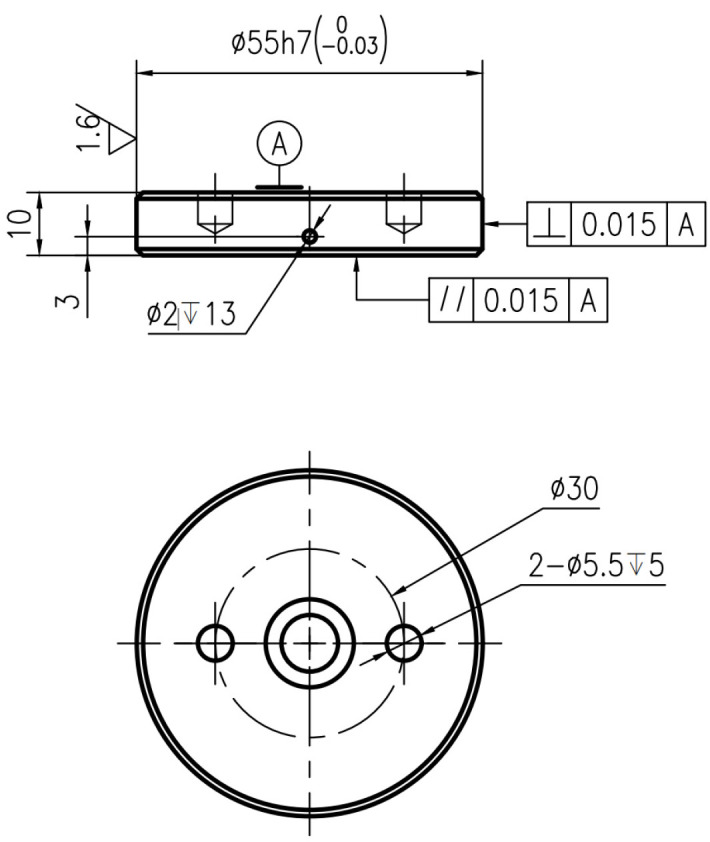
Structure diagram of disc sample (unit: mm).

**Figure 5:**
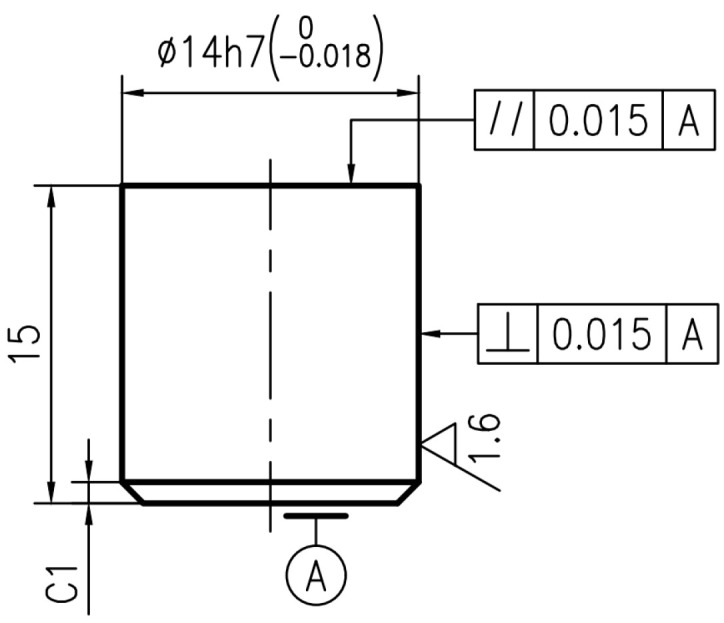
Structure diagram of pin sample (unit: mm).

The brake materials required for the test are all from the high-speed rail site. The thickness of the forged steel brake disc on the site is 16 mm. Combined with the size of the disc fixture of the self-designed testing machine, the forged steel brake disc material is processed into a diameter of 55 mm, a thickness of 10 mm, and the pin specimen is designed to have a diameter of 14 mm, a thickness of 10 mm. Due to the need for the change in temperature of the test disk, a small hole with a depth of 13 mm is opened on the side of the disc and 3 mm from the friction surface. The schematic diagram of the specific disk sample structure is shown in Figure 3, and the physical diagram of the disk sample structure is shown in Figure 4 and Figure 5.

### 2.3. Test Equipment

**Figure 6:**
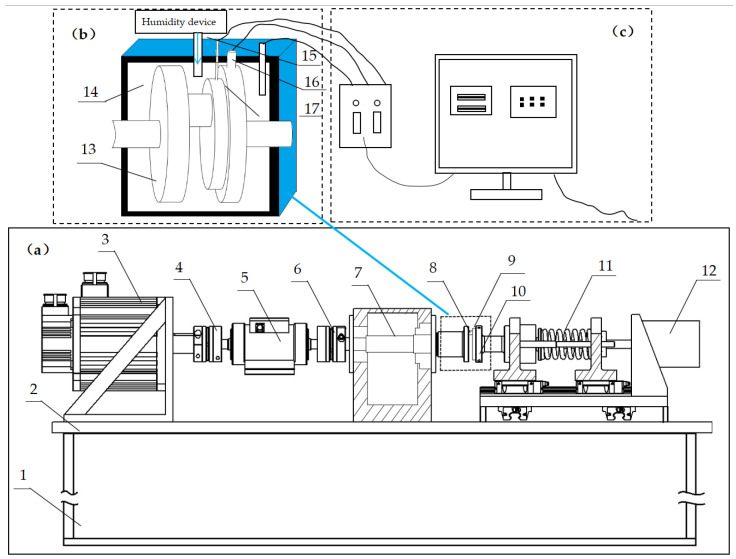
(**a**): Pin disc wear testing machine: (1)–cabinet; (2)–operating platform; (3)–drive motor;(4)–couplings; (5)–torque transducers; (6)–force sensor; (7)–drive shaft; (8)–pin specimens; (9)–disc specimens; (10)–disc fixture: (11)–telescopic shaft; (12)–motor; (**b**): brake friction pair:(13)–pin fixture; (14)–seal the control chamber; (15)–temperature sensor; (16)–high-temperature heating rod; (17)–humidity sensor; (**c**): PLC computer control system.

In this study, a high-temperature pin-disc wear tester (Shandong Province, China) and an ambient humidity device are used to study the friction and wear properties of brake materials. The schematic diagram of the structure of the testing machine is shown in Figure 6. Before the start of the test, the PLC computer parameters are calibrated, and the test study can only be carried out after the data calibration is completed and confirmed. The pin specimen and the disc specimen are first mounted on (8) and (9), respectively, and then fixed. After fixation, the disk specimen can only move in the x-axis direction. We set the test force parameters using computer software. After the parameter setting is complete, it is infinitely motor-loaded (12). The spring and the telescopic catch (11) move slowly to reach the specified test force, and after loading, the pin plate test can set different sizes of contact pressure according to the needs of the test force, and carry out multiple tests. After the test force is loaded, the part of the left pin specimen is responsible for the adjustment of the speed, and the pin plate test can also set different speed tests according to the needs of the test speed. After the computer test parameters, test specimen device and test parameter setting are completed, they are placed in the sealed control room (14), and equipment (16) is used to heat the disc specimen. At the same time, the environment is humidified by the self-upgraded humidity generation device, in the use of temperature sensors (15) and a humidity sensor (17), then the measured values are fed back to the PLC computer.

### 2.4. Test Parameters

Temperature and humidity influence tests on the wear properties of brake disc/brake materials are performed on a multifunctional wear testing machine. The test temperature was 20 °C (normal atmospheric temperature), 100 °C and 200 °C, respectively [22,23,24]. Ambient humidity was normally humid (55%) and high humidity (95%), respectively. During the test, the ambient humidity error is about ±2%. By comparing the experimental and model data, we found that low loads and low sliding speeds do not lead to changes in the friction and wear of the clamps [25,26]. The test was carried out by continuous contact. Forged steel brake disc specimen and powder metallurgy brake specimen’s relative speed is 0.5 m/s, which is converted to a relative sliding speed of 318 r/min. The contact stress is 1.0 MPa, which translates to a test force of about 153 N. The error of the test force is ±2 N. The test time is 120 min, each set of trials is repeated three times. The specific test protocol is shown in Table 2.

### 2.5. Experimental Process

The experimental work in this study is divided into the following steps.

(1) Processing of materials: the brake disc/pad materials brought back from the high-speed railway site are processed separately. At the same time, the brake disc is polished with a polishing machine, so that the roughness of the friction surface is about 0.1 µm; at the same time, the friction surface of the brake pad is polished with 180#, 600# and 1000# sandpaper in order to provide the surface of the brake disc with as much contact with the surface as possible and reduce the gap.
(2) The sample cleaning process: the processed brake disc/pad specimens are exposed to an ultrasonic cleaning machine for cleaning. The cleaning time is 15~20 min. After cleaning work is completed, blowers are used to dry the surface, and the specimens are placed in the drying cabinet for 6 h. Bare hands are not permitted to come into contact with the specimen during the cleaning process; the aim of this is to avoid the grease on the hands adhering to the specimen. The subsequent weighing process and the test process provide the impact.
(3) Weighing of specimens: For the dried specimens, we use an electronic balance with accuracy of 0.001 g and a measuring range of 200 g for weighing and measuring, and repeat the weighing five times for each specimen. We take the average value as the pre-test mass of the specimen. In the process of weighing, in addition to avoiding contact with the hands, each specimen should be marked separately to avoid the occurrence of inconsistent data and specimens.
(4) The preparation work before the test mainly includes calibration of the temperature and humidity sensor of the test machine, the inspection of the experimental equipment, the calibration of the test software and the inspection of the heating device and humidification device.
(5) Installation of specimens: the disk specimens and pin specimens are installed on the fixture of the multifunctional wear tester for fixing.
(6) Measurement of test parameters: we turn on the heating device and humidity device, wait until the test parameters are stable for 10 min, then turn off the heating device, and carry out the test according to the test parameters set in advance.
(7) The processing work after the test: after the test is over, we save the friction coefficient curve and the temperature change curve in the disk obtained during the test, and then wait for the specimen to cool down before we disassemble the specimen.
(8) After the test cleaning work: after the tests on brake disc/pad specimens, and after using the ultrasonic cleaning machine for 15~20 min, we dry the specimen and repeat step 3, respectively. We then take the average value as the test specimen quality.

### 2.6. Test Analysis Method

After the test is completed, the damage mechanism of the brake disc/pad is analyzed and explained in four aspects.

(1) Friction coefficient: According to the position of the pin fixture and the center of the disk, the friction radius of the test is 15 mm. In accordance with the software system that comes with the computer, which we use to derive the friction coefficient graph, the specific algorithm is the ratio of the tangential force to the vertical load of the brake disc and brake pad specimens during the process of sliding contact.
(2) Wear rate: Because of the small amount of wear, the wear rate is tested using the most common method—the gravimetrical method, which is based on the weight change of the specimen before and after the test. The wear amount is determined by weighing with an electronic balance. Therefore, to facilitate the comparison of wear behavior, the mass of the brake disc/pad specimen worn during one week of movement is defined as the wear rate (*α*), and the specific formula is shown in 1.

(1) $$\alpha =\frac{\Delta m}{n}$$

where: *α*—Specimen wear rate in µg/r.

  Δ*m*—Change in mass before and after specimen (mass before test–mass after test), unit: µg.
  *n*—Total number of rotations (revolutions * total time), unit: r.

(3) Analysis of the friction surface morphology: we use the Industrial electron microscope (JT-1400B, China) to analyze the type of damage existing on the surface morphology, the evolution of the damage and the causes of the damage formation.
(4) Change in disk surface temperature: using the temperature sensor of the equipment, the change in disk surface temperature was recorded every 2 min to explore the temperature changes.

## 3. Result

### 3.1. Friction Coefficient

**Figure 7:**
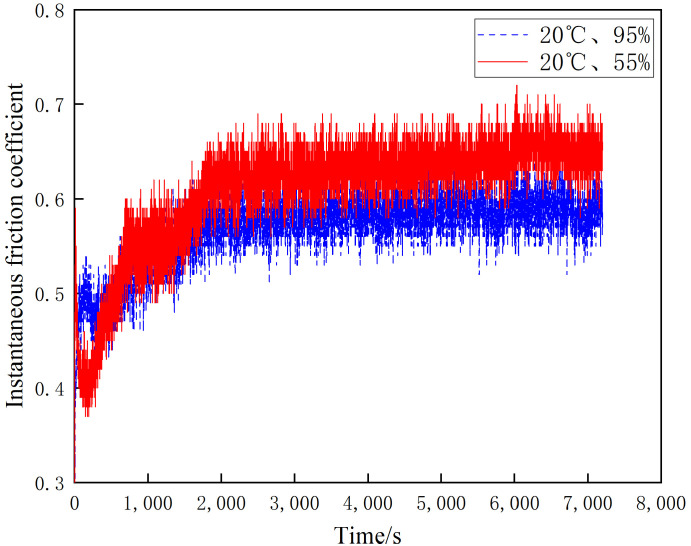
Friction coefficient diagram for different ambient humidity at 20 °C.

**Figure 8:**
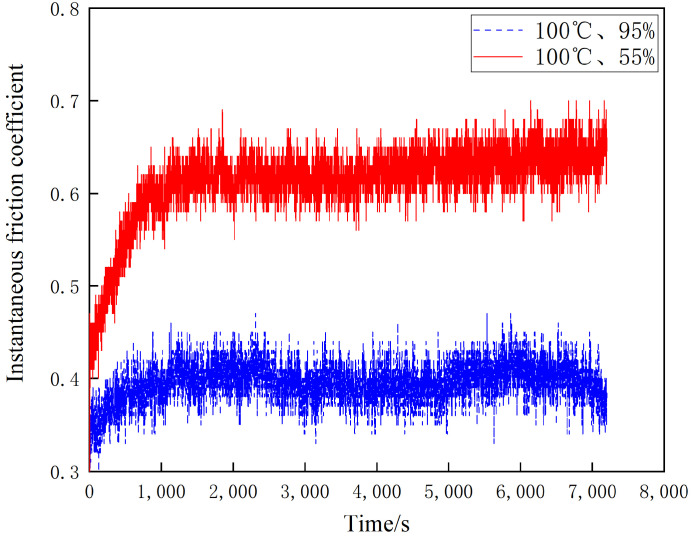
Friction coefficient diagram for different ambient humidity at 100 °C.

**Figure 9:**
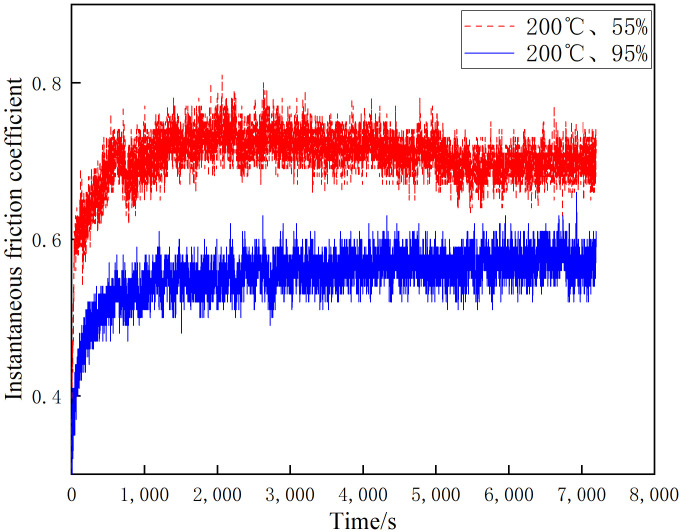
Friction coefficient diagram for different ambient humidity at 200 °C.

Figure 7, Figure 8 and Figure 9 shows the instantaneous friction coefficients of the brake disc/pad specimens at 20 °C, 100 °C and 200 °C and different ambient humidities, respectively. It can be seen from Figure 7 that the instantaneous friction coefficients for both 55% and 95% conditions show a linear and rapid increase during the first 2000 s of wear. After 2000 s, their instantaneous friction coefficients show a slow increase; however, the increase is small. the instantaneous friction coefficients for the 20 °C and 55% conditions are slightly larger than those for the 20 °C and 95% conditions. As shown in Figure 8, the instantaneous friction coefficients at 55% and 95% conditions show a linear increase in the first 1200 s of wear. However, they show an insignificant trend of first decreasing and then increasing after 1200 s. Additionally, it is noteworthy that the instantaneous friction coefficients at 100 °C and 55% are significantly larger than those at 100 °C and 95%. As can be seen in Figure 9, the instantaneous friction coefficients at 55% and 95% conditions show a linear increase during the first 1000 s of wear. After 1000 s, the instantaneous friction coefficients for the 200 °C and 55% conditions tend to decrease slightly, while the 200 °C and 95% conditions maintain a dynamic equilibrium. The instantaneous friction coefficients for the 100 °C and 55% conditions are larger than those for the 100 °C and 95% conditions. It is worth noting that all three data show a similar pattern: the instantaneous friction coefficients under 55% humidity conditions are greater than those under 95% humidity conditions with constant temperature.

### 3.2. Wear Rate

**Figure 10:**
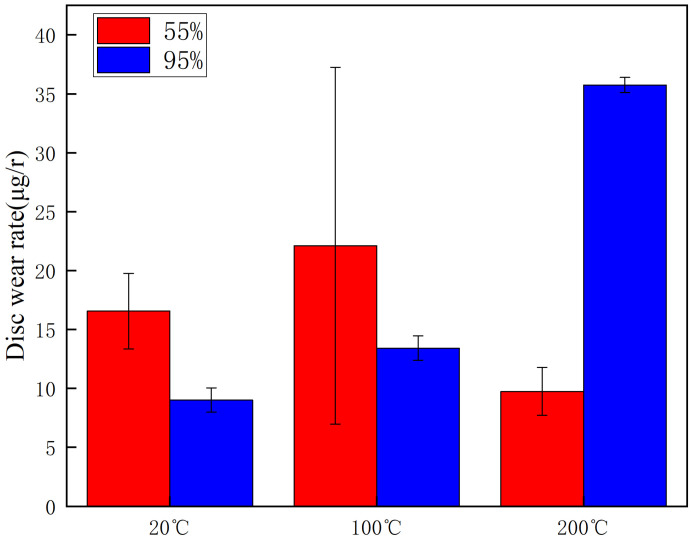
Graph of disk wear rate at different temperatures and ambient humidity.

**Figure 11:**
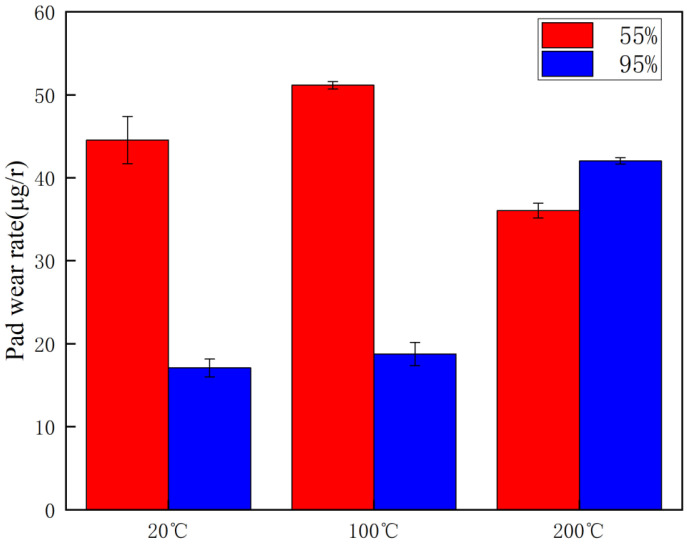
Graph of pad wear rate at different temperatures and ambient humidity.

The wear rates of the brake disc and pad material specimens at different temperatures and ambient humidity are given in Figure 10 and Figure 11, respectively. From Figure 10, it can be seen that the wear rate of the brake disc at 20 °C and 100 °C with medium humidity (55%) is greater than that at high humidity (95%), with a difference of 9 µg/r and 10 µg/r, respectively. Meanwhile, at 200 °C, the wear rate at high humidity (95%) is significantly greater than that at medium humidity (55%). Additionally, it is evident that the disc wear rate reaches 35 µg/r at 200 °C and 95%, which is 60% and 71% higher than the wear rate at 20 °C and 100 °C, respectively, and it can be seen that the wear is most severe at this condition. From Figure 11, it can be seen that the brake pad wear rate shows a similar pattern as the brake disc wear rate, but the disc wear rate is significantly greater than that of the brake disc in the medium-humidity environment (55%). In particular, in the 100 °C condition, the wear rate reaches 51 µg/r.

### 3.3. Surface Morphology

#### 3.3.1. Disk Surface Morphology

**Figure 12:**
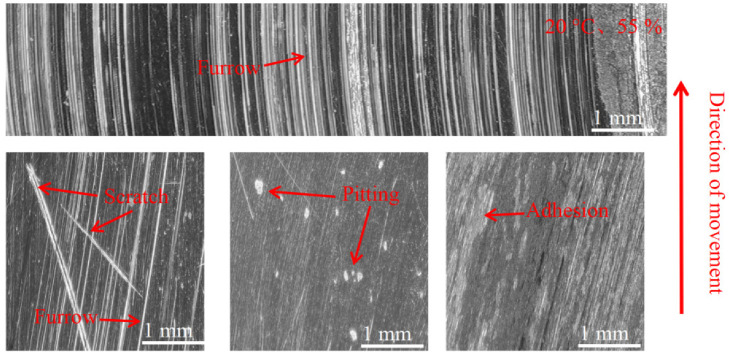
Surface morphology damage map of the disk at 20 °C and 55% ambient humidity.

**Figure 13:**
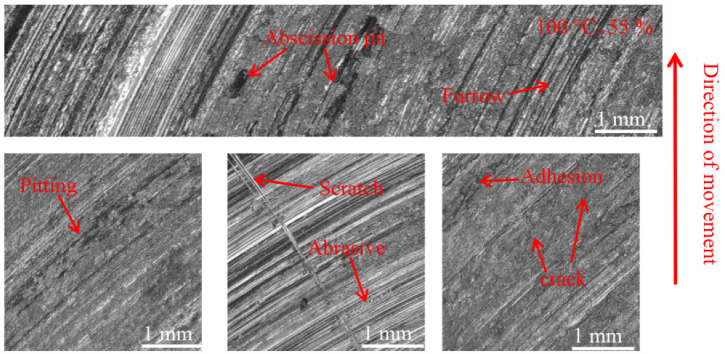
Surface morphology damage map of the disk at 100 °C and 55% ambient humidity.

**Figure 14:**
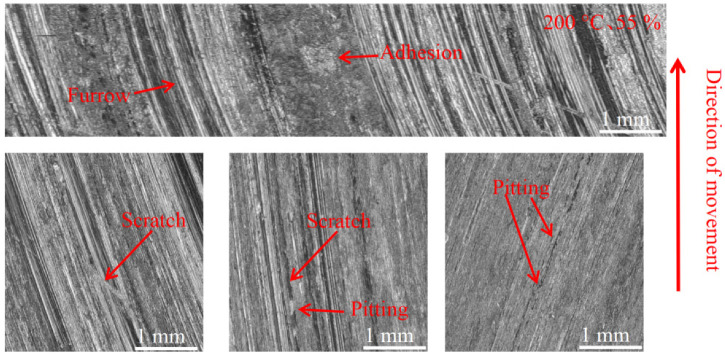
Surface morphology damage map of the disk at 200 °C and 55% ambient humidity.

Figure 12, Figure 13 and Figure 14 show the damage morphology of the disk surface at 55% ambient humidity and different temperatures, respectively. From the figures, it can be seen that the disk surface damage most commonly includes furrows, scratches, pitting, adhesion damage and cracks. The overall disc damage is relatively flat. As can be seen in Figure 12, at 55% ambient humidity, the 20 °C disk damage is mainly characterized by furrows, a large amount of adhesion and a small amount of pitting. As can be seen in Figure 13, at 55% ambient humidity, the 100 °C disk damage is mainly characterized by cracks, a small amount of bonding and a large amount of pitting. In Figure 14, it can be seen that at 55% ambient humidity, the 200 °C disk damage mainly presents as plow furrows and a small amount of adhesion.

**Figure 15:**
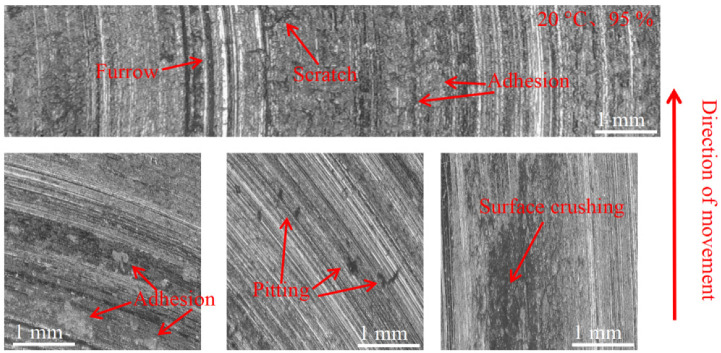
Surface morphology damage map of the disk at 20 °C and 95% ambient humidity.

**Figure 16:**
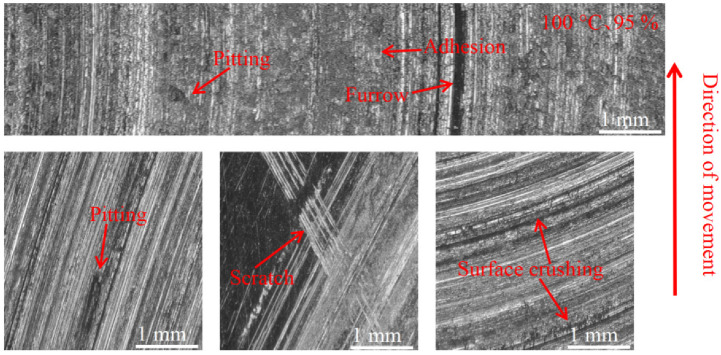
Surface morphology damage map of the disk at 100 °C and 95% ambient humidity.

**Figure 17:**
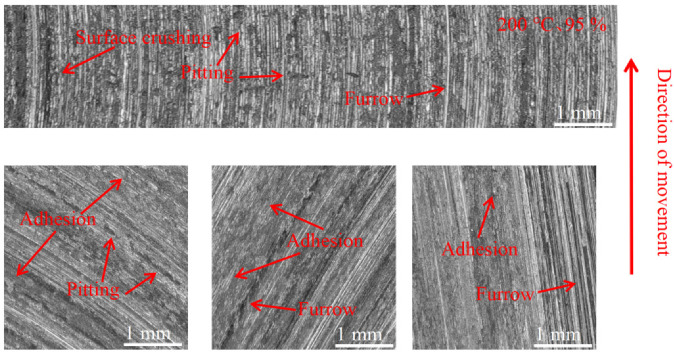
Surface morphology damage map of the disk at 200 °C and 95% ambient humidity.

Figure 15, Figure 16 and Figure 17 show the damage morphology of the disk surface at 95% ambient humidity and different temperatures, respectively. From the figures, it can be seen that the damage on the disk surface mainly takes the form of furrows, scratches, surface crushing and adhesion. From Figure 15, it can be seen that the friction plane is relatively flat compared with Figure 12, and the damage pattern is mainly composed of a large amount of adhesion and a small amount of pitting. From Figure 16, it can be seen that the adhesion gradually reduces and surface crushing is present. As can be seen in Figure 17, the surface roughness of the brake disc increases at 200 °C and 95% ambient humidity, and the surface shows a lot of pitting, surface crushing, a lot of furrows, and small areas of adhesion.

#### 3.3.2. Pad Surface Morphology

**Figure 18:**
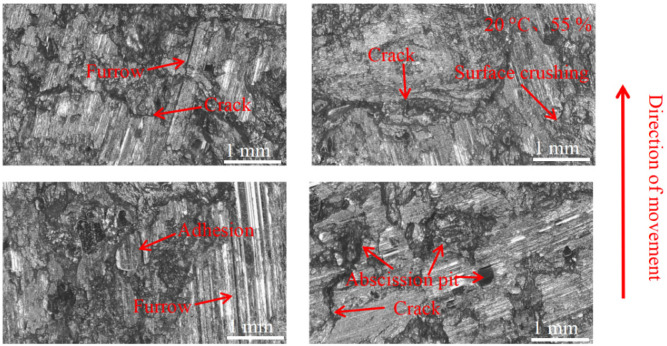
Surface morphology damage map of brake pad at 20 °C and 55% ambient humidity.

**Figure 19:**
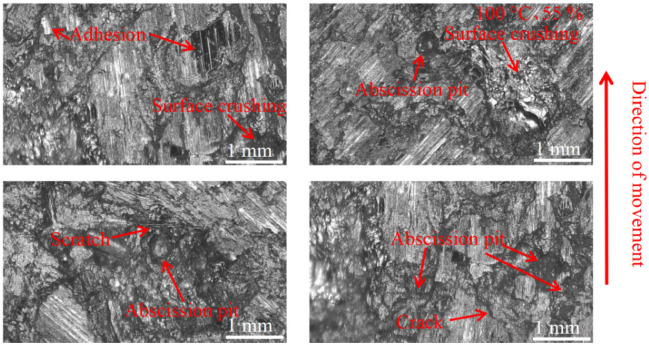
Surface morphology damage map of brake pad at 100 °C and 55% ambient humidity.

**Figure 20:**
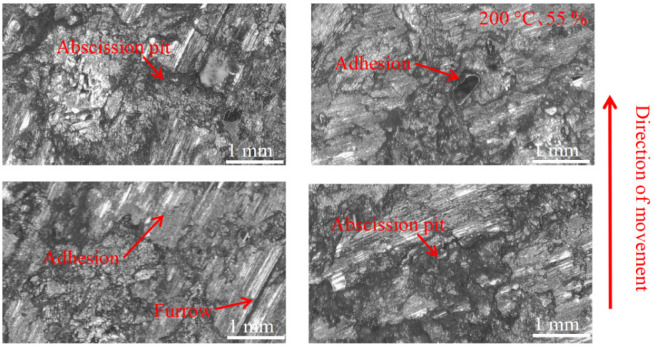
Surface morphology damage map of brake pad at 200 °C and 55% ambient humidity.

**Figure 21:**
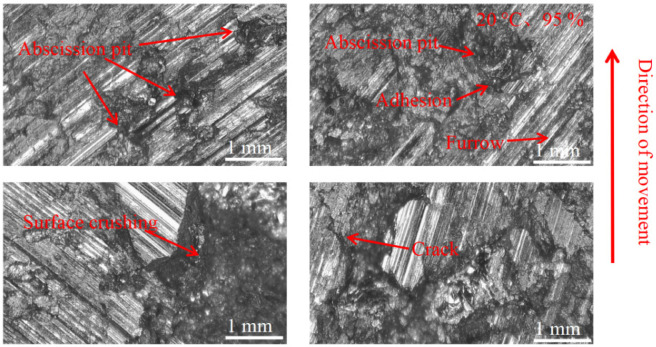
Surface morphology damage map of brake pad at 20 °C and 95% ambient humidity.

**Figure 22:**
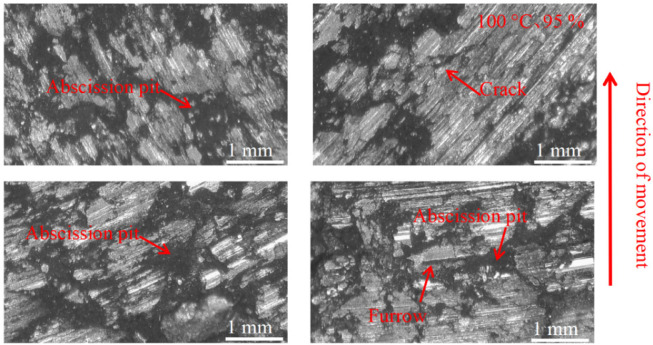
Surface morphology damage map of brake pad at 100 °C and 95% ambient humidity.

**Figure 23:**
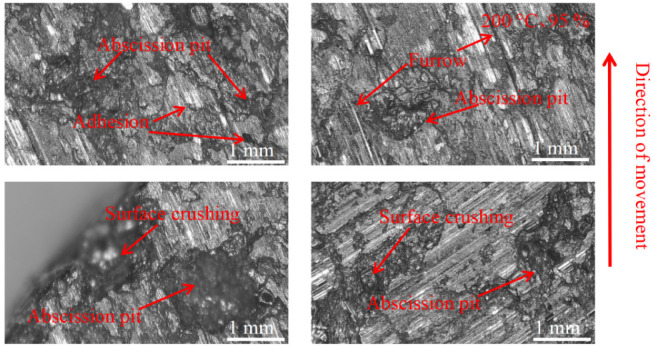
Surface morphology damage map of brake pad at 200 °C and 95% ambient humidity.

As can be seen in Figure 18, Figure 19, Figure 20, Figure 21, Figure 22 and Figure 23, the damage to the brake pads is more severe than the damage to the brake discs under either condition. The brake pad surface damage mainly takes the form of furrows, cracks, adhesion, and abscission pits. It is obvious from Figure 18 and Figure 19 that a large number of abscission pits, cracks and a small amount of adhesive material appear on the brake pad surface, and the friction surface is extremely rough. From Figure 20, it can be seen that the roughness of the friction plane has improved, and although there are a small number of abscission pits, a large amount of adhesive material appears. From Figure 21 and Figure 22, it can be seen that the damage in Figure 22 is the most serious, and there is even a large area of abscission pits at the edge of the brake pad specimen.

### 3.4. Disc Surface Temperature

**Figure 24:**
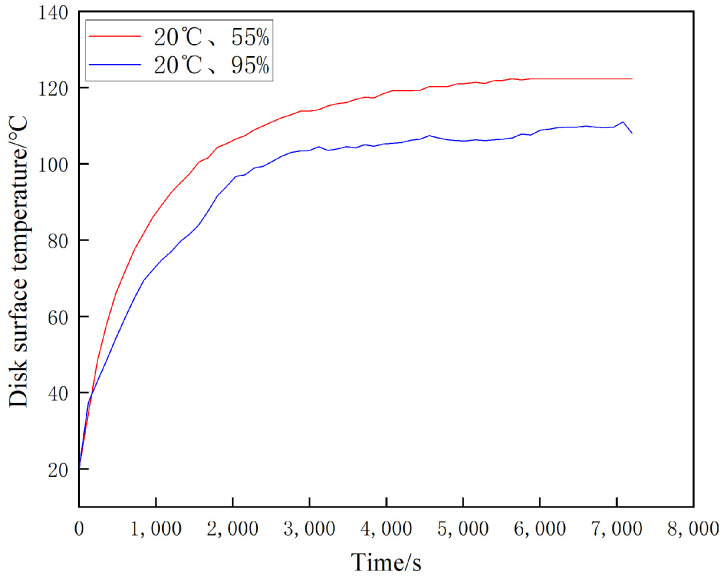
Variation of disc surface temperature at 20 °C under different ambient humidities.

**Figure 25:**
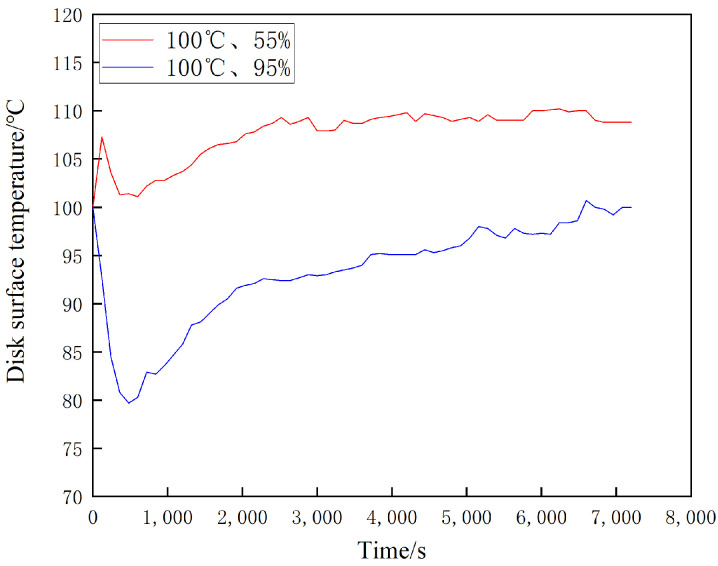
Variation of disc surface temperature at 100 °C under different ambient humidities.

**Figure 26:**
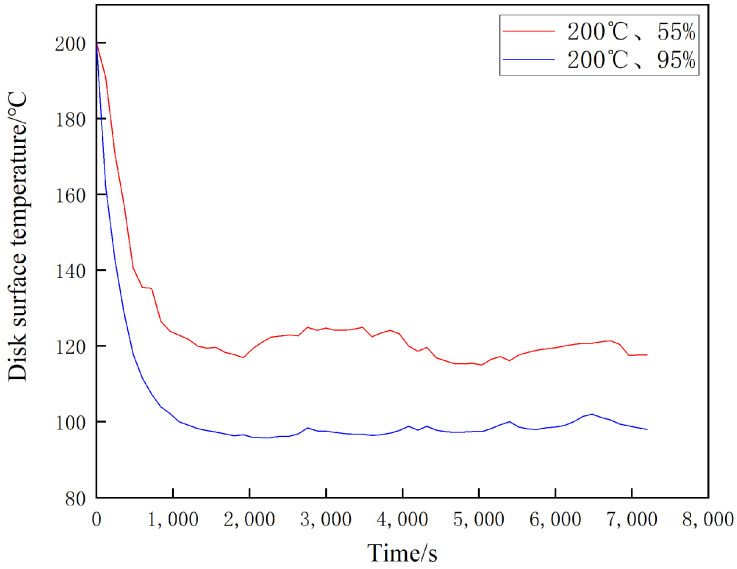
Variation of disc surface temperature at 200 °C under different ambient humidity.

Figure 24, Figure 25 and Figure 26 show the temperature change curves of the disk surface under the same temperature and different ambient humidity conditions, respectively. From Figure 24, it is clear that both temperature rise trends are basically the same. In the first 2000 s when the specimen is rubbed, the disk temperature increases rapidly; during 2000 s–5000 s, the disk temperature grows slowly; and after 5000 s, the disk temperature remains basically the same. The maximum temperature of the disk surface after stabilization at 20 °C and 55% is about 120 °C, while the maximum temperature of the disk surface after stabilization at 20 °C and 95% is about 106 °C, and there is not much difference between the two temperatures at the same time. From Figure 25, it can be found that the fluctuation in the temperature rise curve at 100 °C in the 1200 s of the movement time is large in both 55% and 95% conditions, while 1200 s later shows slow growth. Additionally, the temperature witnessed for both at the same time is very different. It can be seen from Figure 26 that the temperature decreases rapidly during the first 1000 s of the motion time and reaches dynamic equilibrium at 1000 s. The maximum temperature reaches 120 °C after disk stabilization at 200 °C and 55% conditions, and it reaches 100 °C after disk stabilization at 200 °C and 95% conditions.

## 4. Discussion

This test investigates four aspects of the friction and wear performance of the brake material for high-speed rail: instantaneous friction coefficient, wear rate, surface morphology and temperature variation of the disc surface. From the Section 3.1, it is clear that the friction coefficient of medium humidity environment (55%) is larger than that of the high-humidity environment (95%) under the conditions of 20 °C, 100 °C and 200 °C, because the friction surface in the high-humidity environment adsorbs the moisture in the environment and forms a water film on the surface to play the role of lubrication, so the friction coefficient is small in the initial stage of movement [18]. Additionally, according to the temperature of the disk surface, the formation of water film in the disk surface thickness and the maintenance time is different, due to the existence of water film greatly affecting the coefficient of friction. Temperature changes will also affect the length of the water film maintenance time. The high temperature will lead to more rapid evaporation of the water film on the disk surface, which is why the three friction coefficients needed to reach dynamic stability are not consistent. A summary of this can be found in Section 3.2. At 20 °C and 100 °C, the wear rate of 55% ambient humidity is greater than that of 95% ambient humidity. This conclusion is consistent with that of the friction coefficient in Section 3.1, in which the presence of a water film leads to a reduction in the wear rate at 95% ambient humidity. At 200 °C and 95% ambient humidity, the wear rate increases significantly because the high temperature results in the rapid evaporation of the water film and the formation and rupture of the oxide film, and the presence of multiple films leads to an increase in the number of broken particles on the disk surface, resulting in a rapid increase in the wear rate. Due to the different structural composition of the gate material and the disk material, and the presence of a large number of abscission pits in the wear process of the gate specimen, the overall wear rate is greater than that of the disk specimen. Based on the summary presented in Section 3.3, it can be seen that under conditions of 20 °C and 100 °C, the disk damage under 55% ambient humidity is more serious and the friction surface is relatively rough because the friction film will be cut by the shear stress of fine particles after a period of operation. After that, the adhesive will form the friction film. At this time, abrasive wear and adhesive wear are the main considerations, as the humidity increases to 95% due to the existence of water film influencing the friction coefficient [27]. As the humidity increases to 95%, the friction coefficient reduces due to the existence of water film, and although the wear also reduces, the fatigue increases, so abrasive wear, adhesive wear and fatigue wear still occur. Due to the presence of the oxide film, the disk damage at 200 °C and 95% ambient humidity is mainly the result of abrasive wear, adhesive wear and oxidation wear [28]. From the Section 3.4, it can be found that the time point when the temperature equilibrium occurs and the time point when the instantaneous friction coefficient equilibrium occurs basically overlap. This indicates that there is a clear relationship between the factors of disk temperature variation and the friction coefficient. Additionally, 55% ambient humidity is significantly greater than 95% ambient humidity because 95% ambient humidity absorbs some of the heat to promote water evaporation during the temperature variation.

## 5. Conclusions

Main findings:

1. Under certain temperature conditions, the friction coefficient and wear rate decrease with the increase in ambient humidity.
2. With the increase in temperature, the instantaneous friction coefficient and disc surface temperature reach advanced dynamic equilibrium time points at the same time.
3. The brake pad damage is significantly greater than the degree of damage to the brake disc.
4. In 20 °C and 100 °C conditions, 55% ambient humidity brake disc damage mainly takes the form of abrasive wear and adhesive wear. When the humidity increases to 95%, the disc damage comprises abrasive wear, adhesive wear and fatigue wear.
5. Additionally, 5.200 °C and 95% ambient humidity brake disc damage mainly takes the form of abrasive wear, adhesive wear and oxidation wear.
6. The higher the ambient humidity, the lower the surface equilibrium temperature of the brake disc specimen.

In summary, the high-speed rail brake materials show good friction wear performance at 20 °C, 100 °C and 55% ambient humidity. When the humidity increases, the friction coefficient and wear rate of high-speed rail brake materials change significantly, which indicates that the friction and wear performance of brake materials in high-humidity conditions is poor. Therefore, it is recommended to avoid running high-speed trains in a high-humidity environment where possible, and if this cannot be avoided, appropriate measures can be taken to evaporate the disc surface moisture before braking and properly raise the initial temperature of the disc. At the same time, for high-speed trains which need to run in a high-humidity environment for a long time, the amount of brake material overhaul should be increased and a more reasonable overhaul program should be considered.

## Figures and Tables

**Table 1:** Chemical composition of brake disc/brake material (wt/%).

Element	Mn	Cr	Si	C	Ni	Cu	O	S	Fe
Forged steel brake discs	1.02	0.70	0.54	0.42	0.08	-	-	-	BAL
Powder metallurgy brake plates	-	3.47	-	6.75	-	38.85	20.31	1.18	BAL

**Table 2:** Test parameters (repeated three times for each group).

Number	Test Temperature/°C	Test Humidity/%	Contact Stress/MPa	Sliding Speed/m/s	Contact Form
1	20	55	1.0	0.5	Continuous
2	100				
3	200				
4	20	95			
5	100				
6	200				

## References

[1] Ye C., Zheng Y., Lin S. (2022). The Impact of High-Speed Railway Opening on Regional Economic Growth: The Case of the Wuhan–Guangzhou High-Speed Railway Line. Sustainability.

[2] Chen H., Jiang B., Ding S.X. (2022). Data-Driven Fault Diagnosis for Traction Systems in High-Speed Trains: A Survey, Challenges, and Perspectives. Ieee Trans. Intell. Transp. Syst..

[3] Wang Y.P., Ding H.H., Zou Q. (2020). Research progress on rolling contact fatigue of train wheel tread. Surf. Technol..

[4] Zhang C. (2021). Study on Wear and Friction Braking Behavior of EMU Brake Disc/Pad under Low Temperature Environment.

[5] Zhai W.M., Zhao C.F. (2016). Frontiers and Challenges of Modern Rail Transit Engineering Science and Technology. J. Southwest Jiaotong Univ..

[6] Yuan Y. (2017). Study on the Performance of High-Speed Train Braking System.

[7] Lyu Y.Z., Bergseth E., Wahlstrom. J. (2019). A pin-on-disc study on the tribology of cast iron, sinter and composite railway brake blocks at low temperatures. Wear.

[8] Cheng D.D. (2018). Experimental Study on the Effect of Water and Snow on the Friction Coefficient of Brake Friction Pair.

[9] Sun Y. (2020). Study on the Tribological Behavior of High Speed Train Brake Friction Block in Gravel Environment.

[10] Song M.S. (2019). Study on Stress Intensity Factor of Brake Disc Crack Of High-Speed Train.

[11] Song Z.M. (2021). The traction drive of Japanese N700S high-speed train. Foreign Roll. Stock. Technol..

[12] Wang F. (2018). Experimental analysis of cast steel brake discs for high-speed rolling stock. Railw. Veh..

[13] Ji J.Z. (2020). Finite Element Analysis and Optimization of CRH380B EMU Brake Disc.

[14] Shi L.B., Wang F., Ma L. (2018). Study of the friction and vibration characteristics of the braking disc/pad interface under dry and wet conditions. Tribol. Int..

[15] Wang D.W., Wu X., Xiang Z.Y., Mo J.L. (2021). Thermal mechanical coupling characteristics analysis of high-speed train disc brake system. J. Southwest Jiaotong Univ..

[16] Eriksson M., Lundqvist A., Jacobson S. (2001). A study of the influence of humidity on the friction and squeal generation of automotive brake pads. Proc. Inst. Mech. Eng. Part D J. Automob. Eng..

[17] Sun H.Y., Ma Y.M., Chen H., Liu Y., Wu Y., Cen S.B., Wu Q. (2018). Study on High temperature Friction and Wear Behavior of High speed Train Brake Materials. Mechanical.

[18] Rong K.J., Xiao Y.L., Shen M.X. (2021). Influence of ambient humidity on the adhesion and damage behavior of wheel–rail interface under hot weather condition. Wear.

[19] Zhang P. (2020). Substance evolution and wear mechanism on friction contact area of brake disc for high-speed railway trains at high temperature. Eng. Fail. Anal..

[20] Ding S.Y., Ma L., Shi H.B. (2022). Research on the influence of low temperature environment on the fatigue crack growth performance of high-speed train brake disc materials. Mech. Strength.

[21] Sang X.H., Wang X.F., Zhai P.J. (2020). Simulation analysis of train brake disc wear. Mech. Eng. Autom..

[22] Jin Y.X., Wang X.Y., Tong Q.Q. (2014). Study on friction and wear characteristics of T6 SiCp/A356 dry sliding at high temperature. Rare Met. Mater. Eng..

[23] Yao B. (2019). Relationship between Friction Coefficient and Temperature of Friction Pair Of Disc Brake of High-Speed Train.

[24] Liu Y., Wu Y., Ma Y.M. (2019). High temperature wear performance of laser cladding Co06 coating on highspeed train brake disc. Appl. Surf. Sci..

[25] Ma L., Ding S., Zhang C., Zhang M. (2022). Study on the Wear Performance of Brake Materials for High-Speed Railway with Intermittent Braking under Low-Temperature Environment Conditions. Materials.

[26] Lyu Y.Z., Zhu Y., Olofsson U. (2015). Wear between wheel and rail: Apin-on-disc study of environmental conditions and iron oxides. Wear.

[27] Xiao Q., Fang J., Yang Y.H. (2018). Analysis of influence of different temperature and humidity on wheel wear of high-speed train. J. Mech. Eng..

[28] Yuan W.Z., Qiu M., Li X.J. (2010). Study on friction and wear properties of steel/copper pairs in different humidity environment. Lubr. Seal..

